# Intrinsic capacity and mortality in community-dwelling octogenarians: a network analysis from the ilSIRENTE study

**DOI:** 10.1007/s11357-026-02370-0

**Published:** 2026-06-16

**Authors:** Stefano Cacciatore, Bella Bravo, Rola S. Zeidan, Riccardo Calvani, Hyung-Suk Alex Yoon, Matteo Tosato, Andrea Russo, Christiaan Leeuwenburgh, Stephen D. Anton, Emanuele Marzetti, Francesco Landi

**Affiliations:** 1 Department of Physiology and Aging, College of Medicine, Clinical and Translational Science Building, University of Florida, 2004 Mowry Rd, Gainesville, FL 32610 USA; 2 Department of Health Outcomes and Biomedical Informatics, College of Medicine, University of Florida, Gainesville, FL USA; 3 Fondazione Policlinico Universitario “Agostino Gemelli” IRCCS, Largo Agostino Gemelli 8, 00168 Rome, Italy; 4 Department of Geriatrics, Orthopaedics and Rheumatology, Università Cattolica del Sacro Cuore, Largo Francesco Vito 1, 00168 Rome, Italy; 5 Department of Surgery, College of Medicine, University of Florida, Gainesville, FL USA; 6 University of Florida Health Cancer Institute, Gainesville, FL USA; 7 Division of Geriatric Medicine, Department of Medicine, College of Medicine, University of Florida, Gainesville, FL USA

**Keywords:** Intrinsic capacity, Older adults, Mobility, Cognitive function, Mortality

## Abstract

Intrinsic capacity (IC) is a key construct within the healthy ageing framework. However, the correlations among IC domains and their association with health outcomes in very old adults remain unclear. This study aimed to characterise the relationships among IC domains using network analysis and to examine their association with mortality in community-dwelling octogenarians from the Invecchiamento e Longevità nel Sirente (ilSIRENTE) cohort. IC was calculated as a summary score rescaled to a 0–100 range across locomotion, cognition, vitality, psychological capacity, and sensory function, derived from routine home-based geriatric assessments (Minimum Data Set for Home Care instruments and supplementary tests). Assessments were conducted at designated centres, with home visits arranged for individuals unable to travel. The final sample included 319 community-dwelling adults aged ≥ 80 years (mean age 85.4 ± 4.8 years; 67.1% women). Network analysis suggested clustered relationships among vitality, psychological, and cognitive domains, whereas sensory domains appeared more peripheral. Hearing and vision were associated with locomotion, while their association with cognition appeared to be mediated by locomotion. In Cox proportional hazards models, higher locomotion scores were independently associated with lower mortality at 4 years [hazard ratio (HR) 0.86, 95% CI 0.80–0.93] and 7 years (HR 0.87, 95% CI 0.81–0.92). Each 10-point increase in IC was associated with lower mortality at 4 years (HR 0.67, 95% CI 0.58–0.77) and 7 years (HR 0.68, 95% CI 0.61–0.77). Among very old adults, locomotion emerged as a central functional domain linking sensory function, cognition, and survival within the IC framework. IC measures derived from routine assessments may provide a pragmatic approach for risk stratification and targeted interventions to promote healthy ageing.

**Supplementary Information:**

The online version contains supplementary material available at https://doi.org/10.1007/s11357-026-02370-0.

## Introduction

Ageing is associated with progressive declines across multiple systems, leading to increased vulnerability to disability, dependence, and adverse health outcomes [1]. In advanced age, mobility limitations affect more than one third of adults aged 70 years and over half of those aged 80 years or older [2]. Cognitive impairment is also common, affecting approximately 27% of octogenarians and more than 40% of adults aged 85 years or older [3]. Despite these shared risks, trajectories of functional decline in very old age remain highly heterogeneous, with substantial variability across domains [4].

To better capture this complexity, the World Health Organisation (WHO) introduced the concept of intrinsic capacity (IC), defined as the composite of an individual’s physical and mental capacities [5]. IC is operationalised through five core domains: cognition, psychological capacity, locomotion, vitality, and sensory function (hearing and vision). Within the WHO healthy ageing framework, IC reflects functional reserve and the ability to maintain health and independence in later life [5–8].

Although IC domains are conceptually distinct, they rarely deteriorate independently. Cross-sectional studies indicate that impairments in two or more domains coexist in over 50% of adults aged 80 years or older [9]. Importantly, decline across domains does not necessarily occur in parallel, and impairment in one domain may influence others through shared functional and physiological pathways [10]. Nevertheless, most existing studies have examined IC domains separately or have relied on aggregate scores, limiting insight into domain-specific interrelationships and potentially obscuring the underlying structural organisation of IC [11]. In addition, evidence specifically focusing on very old populations remains limited. This population is of particular interest because the accumulation of impairments across multiple domains, combined with survivor effects and heterogeneous ageing trajectories, may result in distinct patterns of inter-domain relationships and differential risk of adverse outcomes compared with younger older adults [12, 13].

Network analysis offers a methodological framework to conceptualise IC as an interconnected system rather than a set of independent domains [10, 14]. Within this context, network approaches may provide a complementary, systems-level perspective on IC by describing patterns of inter-domain connectivity, although their application in this field remains relatively limited and largely exploratory.

The aims of this study were threefold: (1) to characterise the inter-domain relationships of IC using network analysis in a prospective cohort of community-dwelling octogenarians from the Aging and Longevity in the Sirente Geographic Area (ilSIRENTE) study; (2) to examine associations of sensory and psychological domains with locomotion and cognition using regression models and exploratory mediation analyses informed by the network findings; and (3) to evaluate the association between IC and all-cause mortality using both an unweighted IC summary score and a network-weighted IC score. By integrating exploratory network modelling with conventional regression and survival analyses, we aimed to generate hypotheses on the structural organisation of IC and its potential clinical relevance in very old adults.

## Methods

### Study design and population

**Fig. 1:**
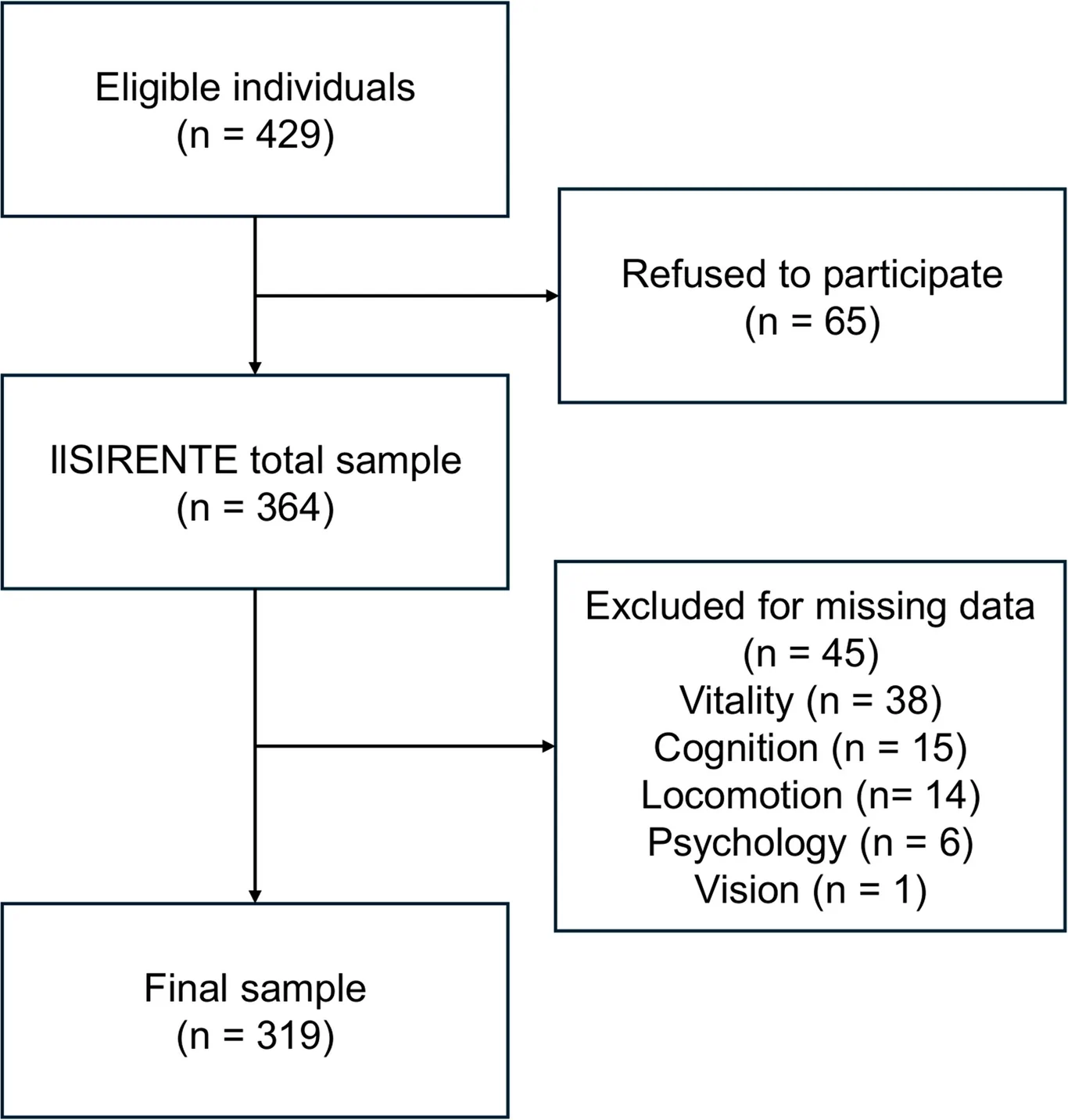
Flowchart of sample selection

This is a prospective cohort study using data from the ilSIRENTE study, a population-based observational study of community-dwelling adults aged 80 years or older living in the Sirente area (Abruzzo, Italy). Participants underwent standardised home-based clinical and functional assessments [15]. The cohort included all residents born before January 1 st, 1924, and living in the thirteen municipalities of the Sirente area at the time of recruitment. Participants were followed for up to 20 years, with mortality status assessed at multiple follow-up waves through December 2023. Of 429 eligible individuals, 65 declined participation, resulting in an initial sample of 364 participants. For the present analyses, 319 participants with complete data on IC domains were included (Fig. 1). The study was conducted in accordance with the Declaration of Helsinki. The study protocol was approved by the Ethics Committee of the Università Cattolica del Sacro Cuore, and written informed consent was obtained from all participants or their legal representatives.

### Data collection

Baseline assessments were performed between December 2003 and September 2004 through standardised home-based evaluations using the Minimum Data Set for Home Care (MDS–HC), which collects information on sociodemographic characteristics, physical and cognitive function, clinical diagnoses, symptoms, and medication use [16]. Assessments were conducted at designated centres in each municipality, with home visits arranged for individuals unable to travel due to health or logistical constraints. All assessments were conducted by trained physicians involved in the study using structured face-to-face interviews and performance-based tests. Additional information on lifestyle behaviours and physical performance was obtained through supplementary questionnaires and tests.

Current smoking was defined as self-reported tobacco use at least once per week during the year preceding baseline. Excessive alcohol intake was defined as consumption of more than 500 mL of wine per day or an equivalent amount of alcohol. Clinical diagnoses were derived from Section J of the MDS–HC and confirmed by study physicians through participant interviews, medical records, and physical examination. Physical activity was defined as engagement in moderate-intensity leisure or occupational activity for at least two hours per week during the previous year [17]. Body mass index (BMI) was calculated as weight in kilograms divided by height in meters squared (kg/m^2^). Functional status was evaluated using MDS-derived scales for basic (ADL) and instrumental activities of daily living (IADL), both ranging from 0 (complete independence) to 7 (complete dependence) [18, 19].

### Assessment of intrinsic capacity

IC was operationalised as a summary score including locomotion, cognition, vitality, psychological capacity, and sensory function (Supplementary Table S1). Locomotion was assessed using the Short Physical Performance Battery (SPPB), which evaluates standing balance, 4-m gait speed, and repeated chair stands, with scores ranging from 0 to 12 and higher values indicating better performance [20]. Cognition was evaluated using the Cognitive Performance Scale (CPS), derived from five MDS–HC items assessing cognitive status, with scores ranging from 0 (no impairment) to 6 (severe impairment) [21]. Vitality was assessed using the Mini Nutritional Assessment–Short Form (MNA-SF), with scores ranging from 0 to 14, where higher values indicate better nutritional status [22]. Psychological capacity was assessed using the MDS Depression Rating Scale (MDS–DRS), ranging from 0 to 14, with higher scores indicating greater depressive symptom burden [23]. Sensory function was assessed using MDS–HC items evaluating hearing (Section C, item C1) and vision (Section D, item D1). Hearing was rated on a 4-level scale ranging from 0 (hears adequately) to 3 (highly impaired), while vision was rated on a 5-level scale ranging from 0 (adequate vision) to 4 (severely impaired). Higher scores indicate worse sensory function [24]. Psychological, sensory, and cognitive domains were reverse-scored to ensure consistency in the direction of the IC domains.

To ensure comparability across domains, all scores were rescaled to a 0–100 range, with higher values indicating better capacity. The overall IC score was calculated as the mean of the five normalised domain scores [25–27].

### Statistical analysis

Continuous variables are presented as means (standard deviations) and categorical variables as counts and percentages. Correlations among IC domains were examined using partial correlation network analysis. Domain scores were residualised for relevant covariates (age, sex, years of education, physical activity, and number of chronic diseases) prior to network estimation to reduce confounding and better capture domain-specific associations. A Gaussian graphical model was estimated using graphical LASSO regularisation with model selection based on the Extended Bayesian Information Criterion (EBICglasso). This regularisation approach was selected to reduce the risk of overfitting and improve the stability of network estimation in moderate sample sizes [28]. Edges represent partial correlations between domains after conditioning on all other nodes, with edge thickness reflecting association strength. Partial correlations were interpreted as strong (≥ 0.40), moderate (0.25–0.39), weak (0.10–0.24), or negligible (< 0.10) [29]. To further assess the robustness of the network structure, sensitivity analyses were conducted using different EBIC tuning parameters (γ = 0.25, 0.50, and 0.75). Strength centrality was calculated as the sum of absolute edge weights connected to each node. Multivariable linear regression models examined associations of sensory and psychological domains with locomotion and cognition. The selection of regression models was informed by the network analysis, with a focus on domain pairs showing stronger correlations. Domain scores were modelled per 10-point increase to enhance interpretability, and interaction terms between sensory and psychological domains were tested. Results are reported as regression coefficients (β) with 95% confidence intervals (CI). Exploratory mediation analyses assessed whether locomotion mediated the association between sensory function and cognition using nonparametric bootstrapping with 2000 resamples. All-cause mortality was assessed at 4 and 7 years of follow-up. Cox proportional hazards models examined associations between IC and mortality, analysing IC both as a continuous total score and as individual domains entered simultaneously in multivariable models, allowing estimation of their independent associations with mortality while accounting for inter-domain correlations and facilitating comparison of their relative contributions. Hazard ratios (HRs) with 95% confidence intervals (CI) were expressed per 10-point increase. Models were adjusted for age and sex (Model 1), and additionally for years of education and number of chronic diseases (Model 2). Covariates were selected a priori based on their established relationships with both IC and mortality in older adults, as well as to account for key demographic and clinical confounders. Proportional hazards assumptions were assessed using Schoenfeld residuals. In exploratory analyses, a centrality-weighted IC score was constructed by weighting each domain according to its strength centrality from the network analysis, with weights normalised to sum to one (Supplementary Table S2). Model performance was compared using the Akaike Information Criterion and Harrell’s C index for all-cause mortality. All analyses were performed using R version 4.5.2. Mediation analysis was conducted using the “mediation” package [30]. All tests were two-sided with statistical significance set at p < 0.05.

## Results

### Study population

After exclusion of 45 participants with missing data on IC domains, 319 individuals were included in the analysis. Excluded participants were older (mean age 88.9 vs 85.4 years, *p* < 0.001) and had higher ADL (4.71 vs 0.99, *p* < 0.001) and IADL scores (5.76 vs 2.68, *p* < 0.001). Dementia was more frequent among excluded participants (37.8% vs 6.0%, *p* < 0.001), and they were taking more medications (4.16 vs 3.18, *p* = 0.006). No significant differences were observed for sex, education, body mass index, or most chronic conditions (Supplementary Table S3).

Baseline characteristics are shown in Table 1. Mean age was 85.4 (4.8) years and 67.1% of participants were women. Approximately one third lived alone and nearly two thirds reported being physically active. Mean ADL and IADL scores were 1.0 (2.0) and 2.7 (2.4), and the mean SPPB score was 7.0 (3.6). Mean CPS and MDS–DRS scores were 0.8 (1.4) and 1.4 (2.1), respectively. Mean BMI was 25.8 (4.4) kg/m^2^ and mean MNA-SF score was 12.4 (1.9). Severe hearing and vision impairment were present in 45.8% and 58.8% of participants, respectively. The mean IC score was 80.5 (14.2). Participants had a mean of 2.1 (1.3) chronic diseases and were taking a mean of 3.2 (2.2) medications.

**Table 1:** Characteristics of the study population

	Total sample (*N* = 319)
Age, years	85.4 (4.8)
Sex, female	214 (67.1%)
Education, years	5.1 (1.7)
Living alone	101 (31.7%)
Alcohol abuse	40 (12.5%)
Smoking	8 (2.5%)
Physically active	204 (63.9%)
ADL score (MDS-based, 0–7)	1.0 (2.0)
IADL score (MDS-based, 0–7)	2.7 (2.4)
BMI, kg/m^2^	25.8 (4.4)
MNA − SF total score (0–14)	12.4 (1.9)
SPPB summary score (0–12)	7.0 (3.6)
CPS score (0–6)	0.8 (1.4)
MDS − DRS score (0–14)	1.4 (2.1)
*Hearing impairment*	
Absent	6 (1.9%)
Mild/moderate	167 (52.4%)
Severe	146 (45.8%)
*Vision impairment*	
Absent	11 (3.4%)
Mild/moderate	120 (37.6%)
Severe	188 (58.8%)
Intrinsic capacity score (0–100)	80.5 (14.2)
Coronary heart disease	37 (11.6%)
Chronic heart failure	16 (5.0%)
Dementia	19 (5.9%)
Diabetes	66 (20.7%)
Chronic obstructive pulmonary disease	44 (13.9%)
Parkinson disease	5 (1.6%)
Cancer	16 (5.0%)
Depression	82 (25.7%)
Number of chronic diseases	2.1 (1.3)
Number of medications	3.2 (2.2)

Data are reported as means (standard deviations) and absolute numbers (%) for continuous and categorical variables, respectively. Physical activity was defined as self-reported engagement in moderate-intensity leisure or occupational activities for at least two hours per week during the previous year. Abbreviations: *BMI* body mass index, *CPS* cognitive performance scale, *MDS* Minimum Data Set, *MDS − DRS* Minimum Data Set − Depression Rating Scale, *SPPB* short physical performance battery

### Correlations among intrinsic capacity domains

**Fig. 2:**
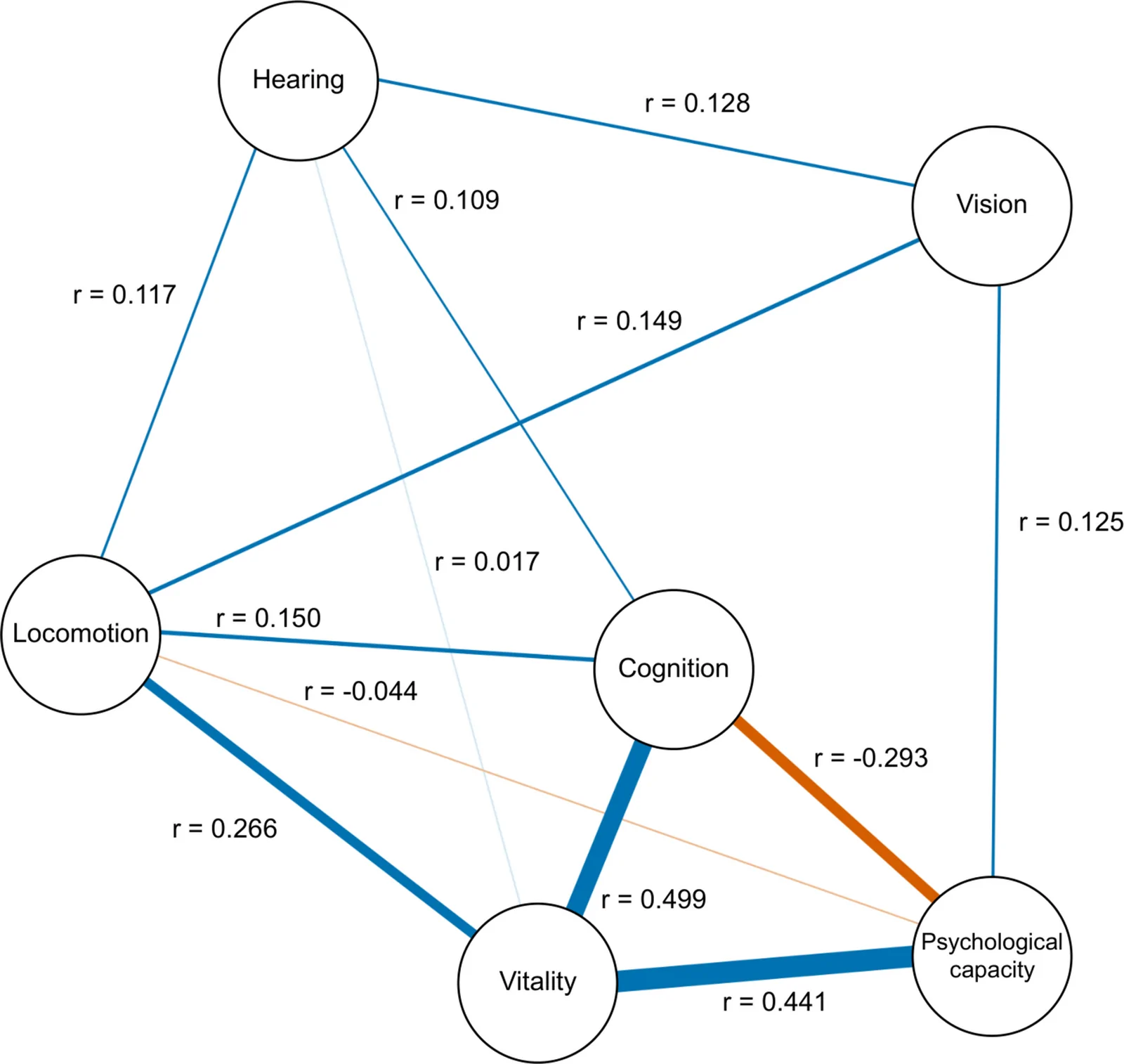
Adjusted partial correlation network of intrinsic capacity domains. Edges represent partial correlation coefficients estimated from domain scores residualised for age, sex, years of education, physical activity, and number of chronic conditions. Edge thickness is proportional to the strength of the association, while blue and orange edges indicate positive and negative correlations, respectively. Only edges with an absolute value ≥ 0.10 are displayed

Network analysis (Fig. 2) identified several non-zero partial correlations among IC domains. Vitality showed moderate positive correlations with the psychological (r = 0.50) and cognitive domains (r = 0.44), whereas a negative association was observed between cognitive and psychological domains (r = − 0.29). Locomotion was positively associated with vitality (r = 0.27) and showed weaker associations with cognition (r = 0.15) and vision (r = 0.15). All reported edges correspond to non-zero partial correlations retained by the EBICglasso model (γ = 0.50). Similar network structures were observed using alternative tuning parameters (γ = 0.25 and γ = 0.75).

### Associations of sensory and psychological domains with locomotion and cognition

In multivariable linear regression models with locomotion as the outcome (Table 2A), higher hearing and vision domain scores were independently associated with better locomotion in the fully adjusted model (hearing: β = 0.49; 95% CI, 0.10 to 0.88; *p* = 0.014; vision: β = 0.43; 95% CI, 0.09 to 0.77; *p* = 0.013 per 10-point increase). The psychological domain was not associated with locomotion, and no significant interaction between vision and hearing domains was observed. Older age, female sex, and a higher number of chronic diseases were associated with lower locomotion scores. In models with cognition as the outcome (Table 2B), sensory and psychological domains were not significantly associated with cognitive performance after adjustment, although a borderline association was observed for hearing (β = 0.41; 95% CI, − 0.01 to 0.84; *p* = 0.059).

**Table 2:** Multivariable associations of sensory and psychological domains with locomotion and cognition

**A) Outcome: locomotion**	**Unadjusted**		**Model 1**		**Model 2**	
	**β (95% CI)**	***p***	**β (95% CI)**	***p***	**β (95% CI)**	***p***
Intercept	−4.10 (− 7.08; − 1.12)	0.007*	6.46 (− 1.92; 14.9)	0.132	11.6 (2.97; 20.2)	0.009*
Vision domain	0.50 (0.16; 0.83)	0.004*	0.46 (0.11; 0.81)	0.011*	0.43 (0.09; 0.77)	0.013*
Hearing domain	0.66 (0.29; 1.03)	< 0.001*	0.55 (0.15; 0.95)	0.008*	0.49 (0.10; 0.88)	0.014*
Psychological domain	0.40 (0.19; 0.62)	< 0.001*	0.31 (0.10; 0.53)	0.005*	0.13 (− 0.10; 0.36)	0.258
Vision × Hearing	− 0.04 (− 0.09; 0.00)	0.057	− 0.04 (− 0.08; 0.01)	0.127	− 0.03 (− 0.08; 0.01)	0.142
Age (per year)			− 0.10 (− 0.17; − 0.02)	0.016*	− 0.12 (− 0.20; − 0.05)	0.002*
Female sex			− 1.26 (− 1.89; − 0.63)	< 0.001*	− 1.27 (− 1.88; − 0.66)	< 0.001*
Education					0.07 (− 0.07; 0.21)	0.326
Number of chronic diseases					− 0.47 (− 0.72; − 0.22)	< 0.001*
**B) Outcome: cognition**	**Unadjusted**		**Model 1**		**Model 2**	
	**β (95% CI)**	***p***	**β (95% CI)**	***p***	**β (95% CI)**	***p***
Intercept	4.83 (1.44; 8.22)	0.006*	7.95 (0.14; 15.7)	0.047*	12.2 (4.26; 20.1)	0.003*
Vision domain	0.29 (− 0.12; 0.69)	0.167	0.26 (− 0.15; 0.68)	0.215	0.25 (− 0.17; 0.66)	0.244
Hearing domain	0.49 (0.08; 0.90)	0.019*	0.45 (0.02; 0.87)	0.042*	0.41 (− 0.01; 0.84)	0.059
Psychological domain	− 0.02 (− 0.17; 0.12)	0.757	− 0.01 (− 0.16; 0.15)	0.933	− 0.14 (− 0.33; 0.05)	0.146
Vision × Hearing	− 0.03 (− 0.08; 0.02)	0.217	− 0.03 (− 0.08; 0.02)	0.264	− 0.03 (− 0.08; 0.02)	0.287
Age (per year)			− 0.03 (− 0.11; 0.04)	0.335	− 0.06 (− 0.13; 0.01)	0.104
Female sex			0.11 (− 0.48; 0.70)	0.715	0.07 (− 0.52; 0.65)	0.824
Education					0.00 (− 0.12; 0.12)	0.971
Number of chronic diseases					− 0.36 (− 0.60; − 0.11)	0.005*

^*^ Indicates statistical significance (p < 0.05)

Model 1: adjusted for age and sex. Model 2: adjusted for age, sex, education, and number of chronic diseases. Regression coefficients (β) are reported per 10-point increase in intrinsic capacity domain scores. Abbreviation: *CI* confidence interval

### Exploratory mediation analysis of sensory function, locomotion, and cognition

In exploratory mediation analyses (Supplementary Figure S1), locomotion partially mediated the association between sensory function and cognition. The indirect effect was statistically significant (ACME = 0.12; 95% CI, 0.06 to 0.19; *p* < 0.001), and the direct effect also remained significant (ADE = 0.16; 95% CI, 0.01 to 0.31; *p* = 0.037). The total effect was significant (0.28; 95% CI, 0.12 to 0.44; *p* = 0.001). The estimated proportion mediated was 0.42 (95% CI, 0.22 to 0.90; *p* = 0.001).

### Survival analysis for 4-year and 7-year mortality

During follow-up, 117 deaths occurred within 4 years and 181 within 7 years. Higher IC, modelled as a continuous score, was associated with lower all-cause mortality at both time points. When IC domains were entered simultaneously, locomotion was the only domain independently associated with mortality. In fully adjusted models, higher locomotion scores were associated with lower mortality at 4 years (HR 0.86, 95% CI 0.80 to 0.93; *p* < 0.001) and 7 years (HR 0.87, 95% CI 0.81 to 0.92; *p* < 0.001) (Table 3). Other domains were not independently associated with mortality. Each 10-point increase in IC was associated with lower mortality at 4 years (HR 0.67, 95% CI 0.58 to 0.77; *p* < 0.001) and 7 years (HR 0.68, 95% CI 0.61 to 0.77; *p* < 0.001). The network centrality-weighted IC score showed similar predictive performance compared with the unweighted score (ΔAIC ≤ 2; Supplementary Table S4).

**Table 3:** Cox proportional hazards models for 4-year and 7-year all-cause mortality according to intrinsic capacity, modeled as a total score and as individual domains, in community-dwelling octogenarians

**A) Timepoint: 4 years**	**Unadjusted**		**Model 1**		**Model 2**	
	**HR (95% CI)**	***p***	**HR (95% CI)**	***p***	**HR (95% CI)**	***p***
*Exposure: Total intrinsic capacity*						
Intrinsic capacity score	0.65 (0.58, 0.73)	< 0.001	0.66 (0.59, 0.75)	< 0.001	0.67 (0.58, 0.77)	< 0.001
Age (per year)			1.04 (1.01; 1.08)	0.014	1.04 (1.00; 1.08)	0.031
Female sex			0.65 (0.44; 0.95)	0.026	0.63 (0.42; 0.92)	0.018
Education					0.93 (0.83; 1.05)	0.265
Number of chronic diseases					1.01 (0.86; 1.17)	0.926
*Exposure: Intrinsic capacity domains*						
Cognition	0.93 (0.86, 1.01)	0.078	0.95 (0.87, 1.03)	0.201	0.95 (0.87, 1.03)	0.201
Psychological	1.16 (1.00; 1.36)	0.057	1.13 (0.96; 1.32)	0.128	1.13 (0.96; 1.32)	0.128
Locomotion	0.86 (0.80; 0.93)	< 0.001	0.86 (0.80; 0.93)	< 0.001	0.86 (0.80; 0.93)	< 0.001
Vitality	0.87 (0.73; 1.05)	0.145	0.85 (0.70; 1.02)	0.082	0.85 (0.70; 1.02)	0.082
Vision	0.99 (0.92; 1.07)	0.885	1.01 (0.94; 1.09)	0.792	1.01 (0.94; 1.09)	0.792
Hearing	0.95 (0.88; 1.02)	0.132	0.97 (0.90; 1.04)	0.420	0.97 (0.90; 1.04)	0.420
Age (per year)			1.04 (1.00; 1.07)	0.056	1.04 (1.00; 1.08)	0.034
Female sex			0.61 (0.41; 0.92)	0.019	0.61 (0.40; 0.92)	0.019
Education					0.92 (0.81; 1.04)	0.199
Number of chronic diseases					1.11 (0.94; 1.31)	0.216
**B) Timepoint: 7 years**	**Unadjusted**		**Model 1**		**Model 2**	
	**HR (95% CI)**	***p***	**HR (95% CI)**	***p***	**HR (95% CI)**	***p***
*Exposure: Total intrinsic capacity*						
Intrinsic capacity score	0.68 (0.61, 0.75)	< 0.001	0.69 (0.62, 0.77)	< 0.001	0.68 (0.61, 0.77)	< 0.001
Age (per year)			1.06 (1.03, 1.10)	< 0.001	1.06 (1.03, 1.09)	< 0.001
Female sex			0.57 (0.42, 0.77)	< 0.001	0.55 (0.40, 0.75)	< 0.001
Education					0.95 (0.87, 1.04)	0.284
Number of chronic diseases					0.97 (0.85, 1.10)	0.599
*Exposure: Intrinsic capacity domains*						
Cognition	0.93 (0.87, 0.99)	0.026	0.94 (0.88, 1.01)	0.092	0.95 (0.89, 1.02)	0.130
Psychological	1.16 (1.02, 1.32)	0.024	1.11 (0.97, 1.26)	0.122	1.14 (0.98, 1.31)	0.089
Locomotion	0.87 (0.82, 0.93)	< 0.001	0.86 (0.81, 0.92)	< 0.001	0.87 (0.81, 0.92)	< 0.001
Vitality	0.91 (0.78, 1.06)	0.214	0.89 (0.76, 1.04)	0.157	0.88 (0.75, 1.03)	0.118
Vision	0.98 (0.92, 1.05)	0.595	1.00 (0.94, 1.07)	0.992	1.00 (0.94, 1.06)	0.946
Hearing	0.94 (0.89, 1.00)	0.037	0.97 (0.92, 1.03)	0.396	0.98 (0.93, 1.04)	0.566
Age (per year)			1.05 (1.02, 1.09)	< 0.001	1.06 (1.02, 1.09)	< 0.001
Female sex			0.54 (0.39, 0.76)	< 0.001	0.54 (0.38, 0.75)	< 0.001
Education					0.95 (0.86, 1.04)	0.238
Number of chronic diseases					1.04 (0.91, 1.20)	0.552

Model 1: adjusted for age and sex. Model 2: adjusted for age, sex, education, and number of chronic diseases. HRs (95% CIs) are expressed per 10-point increase. Abbreviation: *CI* confidence interval, *HR* hazard ratio

## Discussion

In this population-based cohort of community-dwelling adults aged 80 years and older, IC showed a structured pattern of correlated domains. Vitality, psychological, and cognitive domains formed a closely connected cluster, whereas sensory domains were more weakly integrated. Sensory function was associated with locomotion but not cognition, and mediation analyses suggested that locomotion may partly account for the link between sensory function and cognitive performance. Higher overall IC was associated with lower all-cause mortality at 4 and 7 years, with locomotion emerging as the only domain independently associated with survival. Network-derived weighting of IC domains did not improve mortality prediction compared with a simple unweighted score.

Our findings are consistent with the conceptualisation of IC as an interconnected system of functional domains rather than a set of independent components. The WHO defines IC as the composite of an individual’s physical and mental capacities, emphasising interactions among locomotion, cognition, vitality, psychological, and sensory domains [31, 32]. Previous studies using structural equation modelling (SEM) and large longitudinal cohorts have similarly shown that IC domains form an interrelated multidimensional construct associated with functional decline and disability [33–35]. Network analysis should be interpreted as a complementary approach to describe patterns of inter-domain connectivity rather than to infer structural relationships.

Our network analysis identified structured inter-domain relationships even among very old adults. In particular, vitality, psychological function, and cognition showed strong internal connections, suggesting closely linked biological and behavioural processes in advanced age. This pattern is consistent with evidence from large real-world and longitudinal studies showing that IC domains cluster into multidomain profiles associated with different levels of frailty and functional limitation [36]. In particular, data from the ICOPE-Care program, including more than 20,000 community-dwelling older adults, identified distinct clusters of IC impairment, such as combinations of cognitive, locomotor, and sensory deficits, as well as clusters involving psychological and vitality domains, each associated with progressively higher levels of frailty and limitations in activities of daily living [36, 37]. Similarly, longitudinal analyses from the 10/66 study have demonstrated that IC domains evolve in structured patterns over time, with specific trajectories of deterioration associated with markedly increased risks of frailty, disability, and mortality [37].

In this context, our observations extend prior network-based findings indicating that multidomain impairments frequently co-occur and that inter-domain connectivity increases with ageing and worsening health status [10, 14]. In very old populations, higher network density suggests greater interdependence among domains, reflecting tighter coupling of functional systems rather than stronger individual associations [14]. In parallel, epidemiological studies have shown that associations between physical and cognitive domains strengthen with age, with stronger coupling observed in older age groups and robust correlations between trajectories of decline over time [38, 39]. These findings are further supported by large population-based studies demonstrating increased heterogeneity and variability in IC after the age of 60, alongside progressive decline across multiple domains [40, 41]. This body of evidence provides a framework to interpret the more tightly interconnected domain structure observed in our cohort.

From a clinical perspective, the identification of multidomain patterns may support risk stratification and guide more integrated approaches to assessment and care. Co-occurring impairments across domains suggest that interventions targeting a single domain may be insufficient, whereas multidomain strategies combining physical, cognitive, and psychosocial components may be more appropriate in individuals with clustered impairments, in line with the Integrated Care for Older People (ICOPE) framework and its real-life implementations [42, 43]. Future research should further explore how specific combinations of IC domain impairments can be used to guide personalised multidomain interventions and optimise functional outcomes in very old populations.

Importantly, the network-informed analyses were used to generate hypotheses that were subsequently examined using more conventional approaches. In particular, the potential central role of locomotion within the IC structure was supported by both regression and survival analyses. This approach links exploratory modelling with clinically interpretable results, allowing identification of relevant pathways while preserving analytical transparency. Consistent with this, our findings also indicate that meaningful information on the prognostic relevance of IC can be obtained using simple domain-based measures derived from routine clinical assessments, in line with recent evidence from large real-world cohorts supporting the use of pragmatic IC metrics [44]. While more complex statistical models such as SEM may provide additional insight into the latent structure of IC, simpler metrics may represent a pragmatic alternative, particularly in settings with limited sample size or resources. Accordingly, the network findings should be interpreted as descriptive of conditional associations rather than indicative of causal or structural relationships.

Another notable finding is the potential pathway linking sensory function, locomotion, and cognitive performance. Although sensory domains appeared relatively peripheral within the IC network, both hearing and vision were independently associated with locomotion in multivariable models, and mediation analyses suggested that locomotion may partly account for the association between sensory function and cognition. The estimated proportion mediated was 42%, although with a relatively wide confidence interval, supporting a cautious interpretation consistent with partial mediation. Nonetheless, our results suggest that sensory, mobility, and cognitive domains may interact through structured functional relationships rather than representing isolated components of ageing. Previous epidemiological studies consistently report associations between sensory impairments and both mobility limitations and cognitive decline in older adults [45–49]. Proposed mechanisms include reduced physical activity, decreased social engagement, and shared neurodegenerative or vascular pathways [48, 50]. In addition, sensory impairments may influence balance, gait, and interaction with the environment, potentially contributing to reduced mobility, which has been consistently associated with cognitive decline [51–56]. Within the sensorimotor integration framework, mobility performance reflects the integration of sensory inputs with motor and cognitive processes [46, 57–59]. Although causal interpretations cannot be inferred from cross-sectional data, locomotion may represent a functional pathway through which sensory deficits influence cognitive vulnerability. Longitudinal studies are needed to clarify the temporal dynamics among these domains within the broader IC construct.

The negative partial correlation between cognitive and psychological domains warrants careful interpretation. In this cohort of community-dwelling older adults with relatively low prevalence of overt cognitive impairment and depression, this finding may reflect a combination of measurement and population-related factors rather than a direct inverse relationship between the two domains. First, self-reported psychological measures may be influenced by reduced awareness of cognitive deficits, even in the absence of overt dementia, potentially leading to an underestimation of depressive or anxiety symptoms in individuals with lower cognitive performance [60, 61]. Second, cross-sectional associations may not capture the dynamic and bidirectional relationships between cognition and psychological health, which are known to evolve over time and may manifest differently across stages of ageing [62, 63]. Third, in relatively healthy populations, limited variability and potential floor effects in both cognitive impairment and psychological distress may attenuate or distort expected associations [64, 65]. Finally, neuropsychiatric symptoms in older adults, particularly in the context of cognitive impairment, may not fully overlap with depressive symptomatology as captured by standard depression scales, and may require more specific instruments for accurate assessment [66, 67]. Similarly, the lack of an independent association between sensory domains and cognition in fully adjusted models may reflect a combination of methodological and biological factors. The use of brief MDS–HC sensory items, which may have limited accuracy compared with objective measures, could attenuate observed associations [68, 69]. In addition, collinearity between sensory function, locomotion, and other IC domains, as well as the inclusion of locomotion in the fully adjusted models, may have reduced the apparent independent effect of sensory domains. Consistent with existing literature, the relationship between sensory impairment and cognition may be largely mediated through intermediate pathways such as mobility, physical activity, and social engagement, which may be more evident in longitudinal rather than cross-sectional analyses [70–73].

When domains were considered simultaneously, locomotion was the only IC domain independently associated with mortality at both follow-up time points. This finding is consistent with evidence showing that mobility measures such as gait speed and the SPPB are strong predictors of mortality and adverse outcomes in older adults [74, 75]. Physical performance reflects the integrated function of multiple physiological systems and may represent an indicator of overall physiological integrity and biological ageing [76, 77]. Within the IC framework, locomotion may represent a central domain linking multiple components of IC with survival and may serve as a pragmatic marker of overall functional reserve in very old age. Future research should aim to identify more sensitive markers of locomotor function, as traditional mobility tests may show ceiling effects in younger or higher-functioning populations [78–80]. Emerging digital biomarkers from wearable sensors, including activity fragmentation or lower-limb muscle power, may offer promising tools for earlier detection and monitoring of functional decline [78, 81].

These observations align with the conceptual framework of IC proposed by the WHO within the ICOPE strategy [43, 82]. In this model, preserving IC represents a central objective of health systems, shifting the focus from the identification of deficits toward the maintenance of functional abilities across multiple domains. Within this perspective, locomotion may represent a pragmatic entry point for IC-based assessment and intervention. Mobility-targeted interventions, including structured physical activity programmes, fall prevention strategies, and rehabilitation, have consistently been shown to improve physical performance and functional independence in older adults [83, 84]. At the same time, the lack of added predictive value of network-weighted IC scores suggests that simpler approaches based on normalised domain measures may be sufficient for clinical applications. Characterising trajectories of IC over time may help identify individuals with stable, declining, or improving profiles and inform preventive strategies before the onset of disability.

Several limitations should be acknowledged when interpreting the results of the present study. First, the observational design prevents causal inference, and the mediation analyses should therefore be interpreted as exploratory. Second, although IC was operationalised according to established approaches, different measurement models for IC remain under discussion in the literature. In addition, some degree of overlap between IC domains cannot be excluded, as certain components, particularly within the vitality domain, may capture aspects related to mobility and neuropsychological status. While all domains were derived from validated instruments commonly used in geriatric clinical practice and research, this potential overlap may reflect the complexity of the IC construct, which has been operationalised using different approaches across studies, including latent variable models [11]. Third, participants excluded because of missing data were older and more cognitively impaired, which may limit generalisability of our results to frailer populations. Fourth, the relatively moderate sample size may limit the stability and reproducibility of more complex analytical approaches, including network estimation, despite the use of regularisation techniques. Therefore, findings from the network and mediation analyses should be interpreted as exploratory and require confirmation in larger cohorts. Finally, the network analysis reflects cross-sectional relationships among IC domains at baseline and cannot capture dynamic changes in these relationships over time. Future studies should investigate longitudinal changes in IC domain correlations and their association with trajectories of functional decline, resilience, and clinical outcomes.

## Conclusion

In conclusion, IC in very old adults appears to function as an interconnected system of domains rather than a set of independent capacities. Within this system, locomotion emerged as a central functional domain linking sensory function, cognitive vulnerability, and survival. These findings support the potential importance of mobility as both a marker and a target for interventions aimed at preserving IC and promoting healthy ageing in advanced age, while requiring confirmation in larger and longitudinal studies.

## Supplementary Information

Below is the link to the electronic supplementary material.

Supplementary file1 (DOCX 141 KB)

## Data Availability

The data that support the findings of this study are available on request from the corresponding author. The data are not publicly available due to their containing information that could compromise the privacy of the research participants.
