# Supplementary material for: Intrinsic capacity and mortality in community-dwelling octogenarians: a network analysis from the ilSIRENTE study
Source: GeroScience. 2026 Jun 16;48(6):7489–503. doi: 10.1007/s11357-026-02370-0 (PMC13638906; doi:10.1007/s11357-026-02370-0)
Supplement: Supplementary file 1 [file 11357_2026_2370_MOESM1_ESM.docx]

**Supplementary Table S1.** Operationalisation of intrinsic capacity domains in the ilSIRENTE Study.

| **Domain** | **Assessment tool** | **Variables/items** | **Score range (original)** | **Directionality** | **Rescaling (POMP method)** |
| --- | --- | --- | --- | --- | --- |
| Locomotion | SPPB | Balance, gait speed, chair stands | 0–12 | Higher = better | 0–100 |
| Cognition | CPS (from MDS-HC) | 5 items:   - consciousness (b1); - short-term memory (b2a); - cognition skills for daily decision-making (b4); - expressive communication (c4); - eating (g1ha) | 0–6 | Higher = worse | Inverted and rescaled to 0–100 |
| Psychological capacity | MDS-DRS | 7 mood-related items:   - negative statements (e1a); - persistent anger (e1d); - expression of unrealistic fears (e1f); - repetitive health complaints (e1h); - repetitive anxious complaints (e1i); - sad, pained, worried facial expression (e1l); - crying, tearfulness (e1m) | 0–14 | Higher = worse | Inverted and rescaled to 0–100 |
| Vitality | MNA-SF | Obtained from MDS-HC items and supplemental questionnaires, with measured weight and height:   - recent appetite loss (item k8d); - recent weight loss (item l1a); - mobility (items h2b-h2d); - neuropsychological problems (CPS and MDS-DRS); - BMI (measured weight and height). | 0–14 | Higher = better | 0–100 |
| Sensory function | MDS-HC vision and hearing items | Vision item d1 (0–4);   - 0 = adequate - 1 = impaired - 2 = moderately impaired - 3 = highly impaired - 4 = severely impaired   Hearing item c1 (0–3)   - 0 = hears adequately - 1 = minimal difficulty - 2 = hears in special situations only - 3 = highly impaired | Vision 0–4; Hearing 0–3 | Higher = worse | Hearing: inverted and rescaled to 0–100  Vision: inverted and rescaled to 0–100  Sensory domain: mean of the two rescaled components |

Abbreviations: CPS: Cognitive Performance Scale; BMI: body mass index; MDS-DRS: Minimum Data Set Depression Rating Scale; MDS-HC: Minimum Data Set for Home Care; MNA-SF: Mini Nutritional Assessment–Short Form; POMP: percent of maximum possible; SPPB: Short Physical Performance Battery.

**Supplementary Table S2.** Network-derived strength centrality and corresponding weights used for the construction of the centrality-weighted intrinsic capacity score.

| **Domain** | **Strength centrality** | **Normalized weight** |
| --- | --- | --- |
| Vitality | 1.220 | 0.261 |
| Cognition | 0.994 | 0.212 |
| Psychological | 0.961 | 0.206 |
| Locomotion | 0.726 | 0.155 |
| Vision | 0.402 | 0.086 |
| Hearing | 0.371 | 0.079 |

Strength centrality reflects the sum of the absolute partial correlation coefficients connected to each domain, indicating its overall level of connectivity within the network. Higher values denote domains that are more strongly connected to other domains. Weights were obtained by normalizing strength centrality values to sum to one and were used to construct the centrality-weighted intrinsic capacity score.

**Supplementary Table S3.** Baseline characteristics of participants included in the complete-case analysis compared with those excluded because of missing data.

|  | **Included (n = 319)** | **Excluded (n = 45)** | ***p*** |
| --- | --- | --- | --- |
| Age, years | 85.43 (4.77) | 88.94 (4.56) | <0.001 |
| Sex, female | 214 (67.1) | 30 (66.7) | – |
| Education, years | 5.14 (1.74) | 5.07 (0.45) | 0.785 |
| ADL score | 0.99 (2.04) | 4.71 (2.59) | <0.001 |
| IADL score | 2.68 (2.43) | 5.76 (2.15) | <0.001 |
| BMI, kg/m^2^ | 25.76 (4.43) | 24.64 (5.31) | 0.141 |
| Coronary heart disease | 37 (11.6) | 7 (15.6) | 0.604 |
| Heart failure | 16 (5.0) | 6 (13.3) | 0.063 |
| Diabetes | 66 (21.0) | 13 (29.5) | 0.274 |
| COPD | 44 (13.8) | 5 (11.1) | 0.795 |
| Dementia | 19 (6.0) | 17 (37.8) | <0.001 |
| Parkinson’s disease | 5 (1.6) | 1 (2.2) | – |
| Cancer | 16 (5.0) | 1 (2.2) | 0.650 |
| Number of chronic diseases | 2.13 (1.29) | 2.53 (1.41) | 0.056 |
| Number of medications | 3.18 (2.17) | 4.16 (2.57) | 0.006 |

Data are reported as means (standard deviations) and absolute numbers (%) for continuous and categorical variables, respectively. Abbreviations: ADL: activities of daily living; IADL: instrumental activities of daily living; BMI: body mass index; COPD: chronic obstructive pulmonary disease.

**Supplementary Figure S1.** Mediation analysis examining the association between sensory function and cognition, with locomotion as a mediator. Path coefficients (β) and corresponding p-values are shown for each pathway. Indirect effect (ACME): 0.12 (95% CI: 0.06–0.19; p < 0.001); direct effect (ADE): 0.16 (95% CI: 0.01–0.31; p = 0.037); total effect: 0.28 (95% CI: 0.12–0.44; p = 0.001). Estimates were derived from adjusted linear models.

**Supplementary Table S4.** Comparison of unweighted and network centrality–weighted intrinsic capacity scores for prediction of 4-year and 7-year all-cause mortality.

| **A) Timepoint: 4 years** | **HR (95% CI)** | ***p*** | **AIC** | **C-index** |
| --- | --- | --- | --- | --- |
| Intrinsic capacity score (unweighted) | | | | |
| Unadjusted | 0.65 (0.58, 0.73) | <0.001 | 1254 | 0.682 |
| Model 1 | 0.66 (0.59, 0.75) | <0.001 | 1247 | 0.698 |
| Model 2 | 0.67 (0.58, 0.77) | <0.001 | 1250 | 0.700 |
| Intrinsic capacity score (centrality–weighted) | | | | |
| Unadjusted | 0.64 (0.57, 0.73) | <0.001 | 1255 | 0.680 |
| Model 1 | 0.66 (0.58, 0.75) | <0.001 | 1248 | 0.694 |
| Model 2 | 0.66 (0.58, 0.76) | <0.001 | 1251 | 0.698 |
| **B) Timepoint: 7 years** | **HR (95% CI)** | ***p*** | **AIC** | **C-index** |
| Intrinsic capacity score (unweighted) | | | | |
| Unadjusted | 0.68 (0.61, 0.75) | <0.001 | 1896 | 0.667 |
| Model 1 | 0.69 (0.62, 0.77) | <0.001 | 1870 | 0.693 |
| Model 2 | 0.68 (0.61, 0.77) | <0.001 | 1873 | 0.695 |
| Intrinsic capacity score (centrality–weighted) | | | | |
| Unadjusted | 0.67 (0.61, 0.75) | <0.001 | 1899 | 0.664 |
| Model 1 | 0.69 (0.62, 0.77) | <0.001 | 1872 | 0.692 |
| Model 2 | 0.68 (0.60, 0.77) | <0.001 | 1875 | 0.692 |

Model 1: adjusted for age and sex. Model 2: adjusted for age, sex, education, and number of chronic diseases. HRs (95% CIs) are expressed per 10-point increase. Abbreviation: AIC: Akaike information criterion; CI: confidence interval; HR: hazard ratio.
